# Comparative Analysis of Primary Ovarian Cancer Cells and Established Cell Lines as a New Tool for Studies on Ovarian Cancer Cell Complexity

**DOI:** 10.3390/ijms25105384

**Published:** 2024-05-15

**Authors:** Agnieszka Szyposzynska, Aleksandra Bielawska-Pohl, Maria Paprocka, Julia Bar, Marek Murawski, Aleksandra Klimczak

**Affiliations:** 1 Laboratory of Biology of Stem and Neoplastic Cells, Hirszfeld Institute of Immunology and Experimental Therapy, Polish Academy of Sciences, 53-114 Wroclaw, Poland; agnieszka.szyposzynska@hirszfeld.pl (A.S.); aleksandra.bielawska-pohl@hirszfeld.pl (A.B.-P.);; 2 Department of Immunopathology and Molecular Biology, Wroclaw Medical University, 50-556 Wroclaw, Poland; julia.bar@umw.edu.pl; 3 1st Department of Gynecology and Obstetrics, Wroclaw Medical University, 50-599 Wroclaw, Poland; marek.murawski@umw.edu.pl

**Keywords:** ovarian cancer, primary cells, immortalization, cell line, cancer stem cells, CD133

## Abstract

Primary cancer cells reflect the genetic background and phenotype of a tumor. Immortalized cells with higher proliferation activity have an advantage over primary cells. The aim of the study was to immortalize the primary ovarian cancer (OvCa) cells using the plasmid-carrying human telomerase reverse transcriptase (hTERT) gene and compare their phenotype and biological activity with the primary cells. The primary OvCa3 A and OvCa7 A cells were isolated from the ascitic fluid of two high-grade serous ovarian cancer patients and were characterized using immunocytochemical methods, flow cytometry, real-time RT-PCR, Western blot, metabolic activity, and migratory potential. Both immortalized ovarian cancer cell lines mirrored the phenotype of primary cancer cells, albeit with modifications. The OvCa3 A hTERT cells kept the mesenchymal stem cell phenotype of CD73/CD90/CD105-positivity and were CD133-negative, whereas the cell population of OvCa7 A hTERT lost CD73 expression, but almost 90% of cells expressed the CD133 characteristic for the CSCs phenotype. Immortalized OvCa cells differed in gene expression level with respect to *Sox2* and *Oct4*, which was associated with stemness properties. The OvCa7 A hTERT cells showed higher metabolic and migratory activity and ALDH1 expression than the corresponding primary OvCa cells. Both primary and immortalized cell lines were able to form spheroids. The newly established unique immortalized cell line OvCa7 A hTERT, with the characteristic of a serous ovarian cancer malignancy feature, and with the accumulation of the p53, Pax8, and overexpression of the CD133 and CD44 molecules, may be a useful tool for research on therapeutic approaches, especially those targeting CSCs in ovarian cancer and in preclinical 2D and 3D models.

## 1. Introduction

Ovarian cancer is gathering increased interest among clinicians and scientists due to high mortality and poor prognosis [1]. Serous ovarian cancer, divided into low-grade and high-grade carcinoma, is one of the most frequently diagnosed ovarian epithelial carcinomas [2]. The biological behavior of high-grade serous ovarian cancer, such as rapid growth, high aggressiveness, and chemoresistance, often leads to poor prognosis for patient survival [2]; thus, new therapeutic targets for ovarian cancer treatment are needed [3]. Currently, keen attention is paid to the discovery of rapid screening tests for ovarian cancer and the development of new targeted therapies. For preclinical studies, the ideal source of primary ovarian cancer cells are cells obtained from post-operative tumors in patients with ovarian cancer. However, such cells are not the optimal candidates for use in experimental studies due to the limited time of their biological activity in vitro. In addition to the isolation of cells from post-operative tumor tissue, ovarian cancer cells are sourced from the ascitic fluid. The ascitic fluid accumulates in the peritoneal cavity during pathological processes under excessive inflammation. The ascitic fluid, a common aspirate in patients with advanced ovarian cancer, consists of not only cancer cells, but also normal cells, such as fibroblasts, mesothelial cells, endothelial cells, immune cells, and blood cells. Moreover, the ascitic fluid contains a variety of growth factors, cytokines, chemokines, and extracellular vesicles that are involved in cell-to-cell communication [4,5].

Primary cells, including cancer cells, are a heterogeneous population of cells that consists of different types of cells isolated from tissues and organs. The primary cells reflect a phenotype, fate, and behavior resembling the cells present in the organisms they are derived from [6]. Primary cells are widely used for drug screening, determining the physiological and pathophysiological processes, or analyzing the signaling pathways present in the cells. However, the disadvantages of using primary cells in research are a limited number of obtained cells and a limited number of cell divisions. In contrast, cell lines are homogenous populations that usually consist of one type of cells. The most distinctive advantages of cell lines are unlimited cell numbers and unlimited cell division. However, cell lines may not reflect all the features that the primary cells have.

Various procedures for obtaining cell lines are used in experimental studies. One such procedure is spontaneous immortalization, which occurs during the numerous passages of primary cells (hundreds of cell passages). An example of spontaneous immortalization is the ovarian cancer OvBH-1 cell line derived from the ascitic fluid of a patient with an ovarian clear-cell adenocarcinoma [7] and a high-grade serous ovarian cancer cell line OVPA8 [8]. Another way to obtain a cell line is to immortalize the cells using vectors carrying telomerase or Simian virus 40 (SV40) genes. Telomerase is an enzyme involved in adding guanine-rich repetitive sequences, thus preventing the telomeres from shortening [9]. The cells have a limited number of cell divisions, 50–70, called the Hyflick limit, after which the cells become senescent and die [10]. Stem cells, as well as neoplastic cells, show high telomerase activity, which is why they are capable of unlimited cell division. SV40 is an oncogenic DNA virus. The SV40 gene encodes large and small tumor antigens (T antigens) responsible for regulatory function [11]. Immortalization occurs via the inhibition of retinoblastoma (Rb) and p53 genes by the SV40 large T antigen [12]. Another way to immortalize cells is to employ telomerase activity for the stabilization of telomere length. Telomerase is constitutively activated in most human cancers, but not (or expressed at very low levels) in somatic cells. Alterations in telomere length result in both the suppression and facilitation of malignant transformation by regulating genomic stability and cell lifespan [13]. Primary cells immortalized with human telomerase reverse transcriptase (hTERT) represent a breakthrough in cell biology research because they combine the in vivo nature of primary cells through the preservation of phenotypic properties with the cell lines’ ability to survive continuously in vitro. Moreover, because hTERT, overexpressed in about 90% of tumors, is considered as a direct target of the Wnt/β-catenin signaling pathway, it is involved in the initiation and further progression of cancer [14].

Cancer stem cells (CSCs) are present within the heterogeneous population of cancer cells and are responsible for tumor progression, resistance to chemotherapy and cancer metastasis [15]. CSCs, in addition to the tumor-specific phenotype, are characterized by the presence of markers different from mature cancer cells. The most common marker, CD133 (a cholesterol-binding glycoprotein, prominin), is a marker of stem and progenitor cells [16] that is widely used for the isolation of CSCs from different solid tumors [17]. CD133-positive cells have been documented in ovarian cancer tissues in the context of the expression of p-p53(Ser20) and carcinoma stem cell biomarkers and in the fraction of the OvBH-1 cell line [18]. The co-expression of CD133 and other CSC markers, such as the CD44 transmembrane glycoprotein involved in cell-to-cell interactions [19], the heat-stable antigen CD24 that plays a crucial role in cell adhesion and is involved in tumor metastasis [20], and CD117(c-kit) is observed in studies on ovarian CSCs. Ovarian CSCs play a crucial role in tumor formation and resistance to chemotherapeutics. They also lead to relapse after treatment [21].

Currently, CSCs are extensively studied as a potential target in the treatment of many cancers, including ovarian cancer. In the natural tumor microenvironment, CSCs constitute a small populations of tumor cells usually insufficient for studies on different therapeutic approaches. This study aimed to isolate and characterize primary ovarian cancer cells from the ascitic fluid of patients with high-grade serous ovarian cancer, and afterwards, to establish immortalized human ovarian cancer cell lines with a focus on tumor cells with the CSCs phenotype. The cells were immortalized using a plasmid carrying the human telomerase gene. Finally, the phenotype and functional properties of the immortalized cell lines were characterized to the corresponding primary OvCa cells. 

## 2. Results

### 2.1. Characteristics of Primary Ovarian Cancer Cells and Immortalized Ovarian Cancer Cells

#### 2.1.1. Cytomorphological Assessment

**Figure 1:**
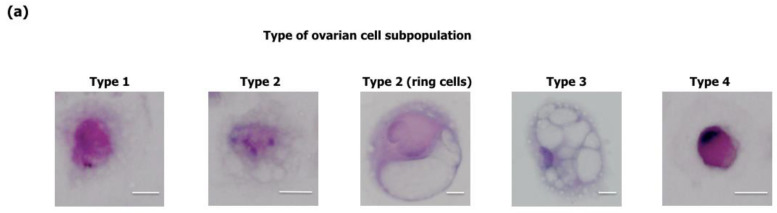

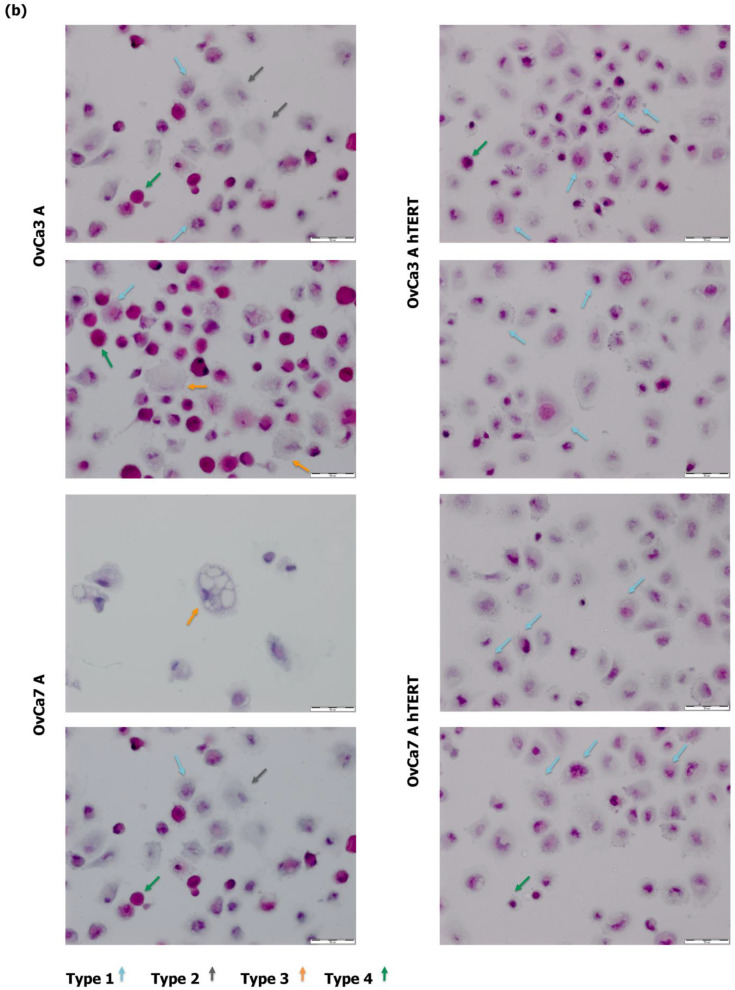

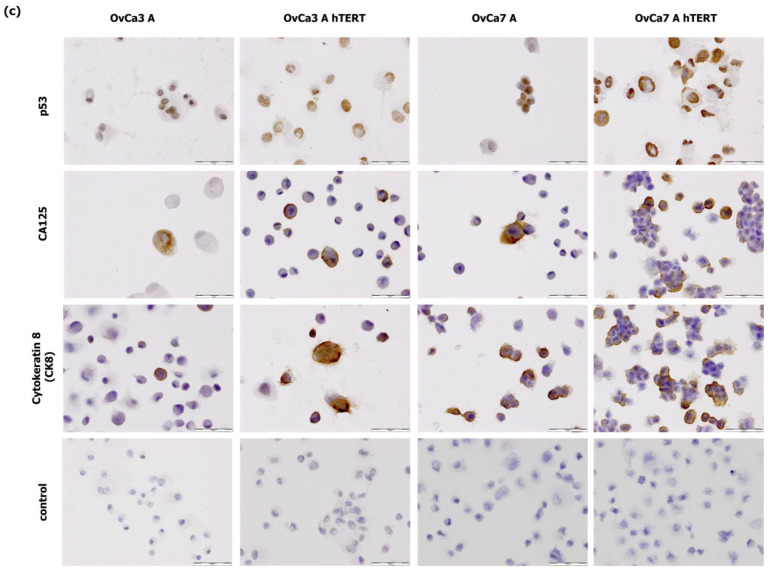
Characteristics of primary ovarian cancer cells and immortalized ovarian cancer cells. Representative images from H and E and immunocytochemical (ICC) staining. (**a**) Representative images of the analyzed types of cells. The bar represents 10 μm. (**b**) The left panel shows images for primary ovarian cancer cells OvCa3 A and OvCa7 A, and the right panel shows images of immortalized ovarian cancer cells OvCa3 A hTERT and OvCa7 A hTERT. The blue arrows indicate type 1 cells, gray arrows indicate type 2 cells, orange arrows indicate type 3 cells, and green arrows indicate type 4 cells. The scale bar represents 50 μm. Abbreviation: H and E—hematoxylin and eosin. (**c**) Representative images from ICC staining. The cell nuclei was stained with hematoxylin (blue), and selected markers were stained with DAB chromogen (brown). The bar represents 50 μm.

Morphological features of the primary cellular composition of the ascitic fluid collected from two patients with poorly differentiated (G3) serous ovarian carcinoma and cells after immortalization are presented in Table 1. The cells were classified according to their morphological features describing malignancy, from cells showing typical features of malignancy to cells with atypia. The primary cellular composition of the two cases consists of four cell subpopulations showing different stages of malignancy corresponding to the stage of cancer. In the first case, designated in Table 1 as type 1 cells, the cells were large, round with scant eosinophilic cytoplasm and indistinct cell borders. The nuclei showed variation in size and shape. Chromatin was dense with hyperchromasia (Figure 1a,b). The nuclear–cytoplasmic ratio was increased in a majority of cells (3:1).

Type 2 cells were large, with indistinct borders and eosinophilic cytoplasm, and contained a single large vacuole defined as “ring cells”, often observed in serous ovarian carcinoma. There were also cells with numerous vacuoles in the cytoplasm (Figure 1a,b). The nuclei were different in size and shape with nucleoli. Type 3 cells were large, with a large cytoplasm containing numerous small vacuoles. The nuclei were different in size (Figure 1a,b). Type 4 cells were medium-sized with a distinct cell border and basophilic cytoplasm (Figure 1a,b). After cell immortalization, two cell populations were classified in both analyzed cases. The first population showed typical malignant features, as found in type 1 cells before immortalization, and these cells were more homogenous than the type 1 subpopulation of primary cells. A characteristic feature of these cells were their very large nuclei (Figure 1a,b). The second type of immortalized cell subpopulations showed morphological features comparable with type 4 primary cells.

To confirm the morphological features of the cells’ malignancy, the p53 immunostaining was performed on both cell lines before and after hTERT immortalization. As showed in Figure 1c, primary cells isolated from ascitic fluid revealed a heterogeneous pattern of p53 immunostaining. Nuclear accumulation of p53 was observed in a different percentage of primary cells. After hTERT immortalization, the number of p53 immunopositive cells with strong nuclear accumulation increased from 40–50% to 90–100% of cells (Figure 1c). The origin of cells form ovarian epithelial tissue was confirmed with the expression of the CA 125 antigen and CK8. Immortalized cells showed a trend towards higher CA 125 and CK8 expression compared to primary ascitic fluid cells (Figure 1c).

#### 2.1.2. Flow Cytometry Analysis

**Figure 2:**
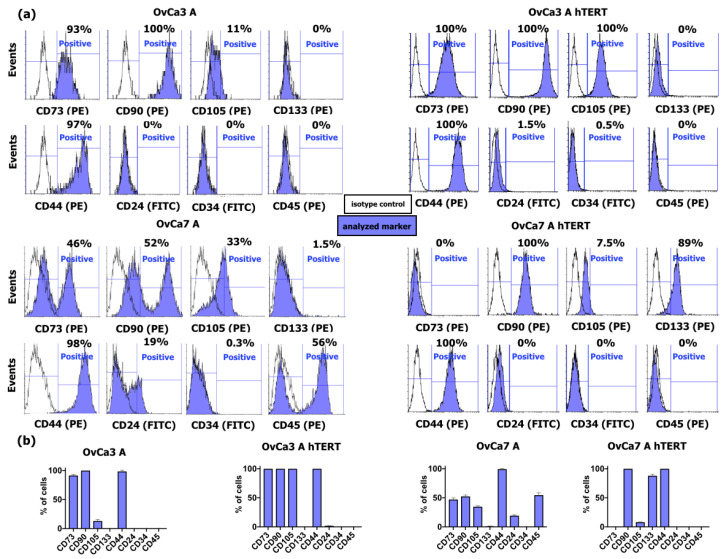
Characteristics of primary ovarian cancer cells and immortalized ovarian cancer cells. (**a**) Representative histograms from flow cytometry analysis. The white histograms refer to isotype controls, and the blue histograms refer to analyzed markers. The left panel shows representative histograms for primary ovarian cancer cells OvCa3 A and OvCa7 A, and the right panel shows representative histograms for immortalized ovarian cancer cells OvCa3 A hTERT and OvCa7 A hTERT. (**b**) The bar charts show the average % of cells positive for selected markers for primary cells and immortalized cells. The data represent mean ± SEM values from three independent experiments.

Flow cytometry analysis was performed to determine the expression of selected mesenchymal stem cell (MSC) markers CD73, CD90, and CD105, marker characteristics for CSCs, such as CD133, CD44, and CD24, as well as marker characteristics for hematopoietic progenitors CD34 and all nucleated hematopoietic cells CD45. The representative histograms are shown in Figure 2a,b.

The primary ovarian cancer cells OvCa3 A were positive for the MSC markers CD73 (91.50% ± 1.50% of the population), CD90 (100.00% of the population), and CD105 (13.00% ± 2.00% of the population). The cells also expressed the CD44 molecule (98.50% ± 1.50% of the population). The analyzed OvCa3 A cells were negative for CSC markers CD133 and CD24 and negative for the hematopoietic markers CD34 and CD45.

In the obtained cell line OvCa3 A hTERT, the entire population of cells was positive for marker characteristics for MSC cells, such as CD73, CD90, and CD105, and for the CD44 marker (each time being 100.00% of the population). A small number of cells expressed the CSC marker CD24 (1.75% ± 0.16% of the population). Moreover, the cells were negative for the selected CSC markers CD133 and markers characteristic for hematopoietic cells CD34 and CD45 (Figure 2a,b). A summary of the results of flow cytometry and immunofluorescence staining is presented on the heatmap (Figure S1).

The primary ovarian cancer cells OvCa7 A expressed the MSC markers CD73 (47.00% ± 1.52% of the population), CD90 (52.33% ± 1.45%), and CD105 (34.66% ± 0.88%). Importantly, 1.40% ± 0.06% of cells were positive for the CSC markers CD133, 19.33% ± 0.88% of the population expressed the CD24 molecule, and 99.30 ± 0.89% of the population were positive for CD44. The primary OvCa7 A cells were negative for CD34 and positive for CD45 (56.67% ± 3.12% of the population) (Figure 2a,b). The immortalized cells OvCa7 A hTERT did not express the CD73 marker. Only 8.00% ± 0.29% of the population expressed the CD105 marker, whereas the entire population expressed the CD90 marker (100.00% ± 0% of the population). Importantly, the cell population was positive for CD133 (88.00% ± 1.52% of the population) and CD44 (100.00% ± 0% of the population), recognized as CSC markers. However, the cells were negative for another CSC characteristic molecule, CD24. Moreover, the OvCa7 A hTERT cells were negative for the CD34 and CD45 markers (Figure 2a,b).

#### 2.1.3. Immunofluorescence Staining and Microscopic Imaging Results

Immunofluorescence staining and microscopic imaging were used to assess the expression of selected cancer cell markers such as p53, Pax8, and cancer-associated fibroblast markers such as platelet derived growth factor receptor alpha (PDGFRα), fibroblast activation protein (FAP), pluripotency-related transcription factors Oct4, Sox2, and Nanog; and markers characteristic for ovarian CSCs, such as CD133, CD44, c-kit, and the marker of epithelial to mesenchymal transition (EMT), Snail molecule. Furthermore, the cytoskeleton of the cells was visualized using F-actin and vimentin staining.

**Figure 3:**
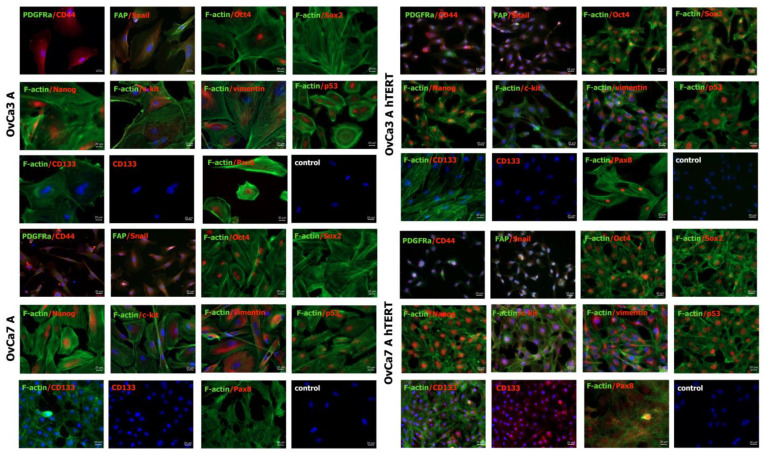
Characteristics of primary ovarian cancer cells and immortalized ovarian cancer cells. Representative fluorescence images. Blue color (DAPI) represents cell nuclei, green color (Alexa Fluor 488) represents selected markers or F-actin, red color (Alexa Fluor 647) represents selected markers. The scale bars represent 20 µm. The left panel shows images for the primary ovarian cancer cells OvCa3 A and OvCa7 A, and the right panel shows images for the immortalized ovarian cancer cells OvCa3 A hTERT and OvCa7 A hTERT. The immunofluorescence staining was performed in duplicate.

The primary ovarian cancer cells OvCa3 A were negative for one of either the CAF markers, PDGFRα, transcription factor Sox2, and CSCs marker CD133. On the other hand, the cells were positive for the pluripotency markers Oct4 and Nanog and adhesion molecule CD44. A weak expression of the EMT marker Snail was observed. Moreover, the cells were positive for the c-kit marker, and some cells expressed the CAF marker FAP. The expression of cancer cell markers p53 and Pax8 was detected. F-actin staining visualized stress fibers across the cells. Furthermore, the presence of intermediate filaments was confirmed with vimentin staining (Figure 3).

In the immortalized OvCa3 A hTERT cells, a weak expression of the CD44 marker was observed. The cells were negative for the FAP molecule and CD133. In contrast to the primary cells, the immortalized cells were positive for all pluripotency markers: Oct4, Sox2, and Nanog. Some cells expressed PDGFRα, and a weak expression of the c-kit marker was observed. The immortalized cells strongly expressed p53 protein and Pax8. The vimentin staining visualized the intermediate filaments around cell nuclei.

As with primary ovarian cancer OvCa3 A, the OvCa7 A cells were negative for Sox2, and positive for Oct4 and Nanog. The OvCa7 A cells were positive for CD44 and Snail protein expression, but negative for the CAFs markers PDGFRα and FAP and CD133 molecule and Pax8. The expression of c-kit, nuclear accumulation of mutated p53 protein, and vimentin was also observed (Figure 3).

In the immortalized OvCa7 A hTERT cells, the nuclear expression of CD44 was observed, and the cells were positive for PDGFRα. The OvCa7 A hTERT cells were negative for FAP, and a weak expression of Snail was detected. As OvCa3 A hTERT cells, the OvCa7 A hTERT cells were positive for all the analyzed markers involved in the maintenance of pluripotency: Oct4, Sox2, and Nanog. Furthermore, the expression of c-kit, CSC marker CD133, and nuclear accumulation of mutated p53 and Pax8 was detected (Figure 3).

#### 2.1.4. Stemness Markers, Cancer Marker Pax8 and CSC Marker CD133 in Immortalized Cell Lines

The RT-PCR was used to determine the presence of transcription factors related to the maintenance of pluripotency: *Oct4*, *Sox2*, protooncogenic molecules *p53*, *p21*, *c-myc*, and CSC marker *CD133*. Both primary cells OvCa3 A and OvCa7 A served as a control for each immortalized cell line, respectively.

**Figure 4:**
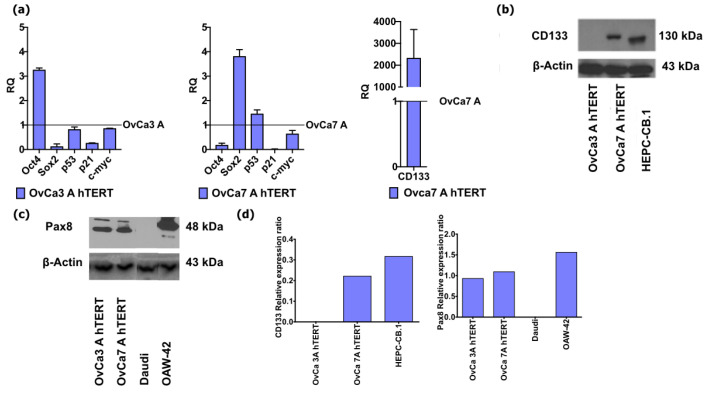
Characteristics of immortalized ovarian cancer cells. (**a**) The bar charts show the relative expression of the pluripotency-related markers *Oct4*, *Sox2*, protooncogenic markers *p53*, *p21*, *c-myc*, and *CD133*. The left bar chart shows the relative expression for OvCa3 A hTERT. The primary ovarian cancer cells OvCa3 A served as a control. The middle and right bar charts show the relative expression for OvCa7 A hTERT, with primary ovarian cancer cells OvCa7 A used as a control. The data represent mean ± SEM values from three independent experiments performed in duplicates. (**b**) Western blot analysis of CD133 molecule expression in immortalized ovarian cancer cell lines. CD133 and β-Actin protein levels were revealed with different sets of specific antibodies in ovarian cancer cell line extracts obtained from lysing cells with the RIPA buffer. Protein extracts from HEPC-CB.1 cells were used as a positive control. CD133—130 kDa and β-Actin—43 kDa expression. Each time, 50 μg/line of total protein was loaded. (**c**) Western blot analysis of Pax8 molecule expression in immortalized cell lines. Pax8 and β-Actin protein levels were revealed with different sets of specific antibodies in cell extracts obtained from lysing cells with the RIPA buffer. Protein extract from OAW-42 cells was used as a positive control, while protein extract from Daudi cells was used as a negative control. Pax8—48 kDa and β-Actin—43 kDa expression. Each time, 50 μg/line of total protein was loaded. The Western blot was performed in one repetition. (**d**) Densitometry analysis of CD133 and Pax8 was performed by using ImageJ software version 1.54f, and is presented as the rate of CD133 and Pax8 molecules expression to β-actin (used as an internal control). The data represent values from one experiment.

In the immortalized OvCa3 A hTERT cells, *Sox2* (RQ 0.130 ± 0.070), *p21* (RQ 0.270 ± 0.001), *p53* (RQ 0.830 ± 0.064), and *c-myc* (RQ 0.869 ± 0.001) expression was downregulated compared to the primary ovarian cancer cells OvCa3 A. Only *Oct4* (RQ 3.263 ± 0.051) was overexpressed in the OvCa3 A hTERT cells (Figure 4a).

In the immortalized OvCa7 A hTERT cells, *Sox2* (RQ 3.819 ± 0.189) and *p53* (RQ 1.466 ± 0.110) expression was upregulated compared to the primary ovarian cancer cells OvCa7 A. In contrast, the expression of *Oct4* (RQ 0.188 ± 0.051), *c-myc* (RQ 0.649 ± 0.092), and *p21* (RQ 0.019 ± 0.004) in the immortalized OvCa7 A hTERT cells was downregulated (Figure 4a).

The expression of CD133 was undetermined in primary OvCa3 A and in immortalized OvCa3 A hTERT. An important observation was that the relative expression of CD133 in OvCa7 A hTERT, which was (RQ 2334 ± 922) over two thousand times higher compared to the primary OvCa7 A cells. To confirm the expression of the CD133 protein, Western blot analysis was performed in the two analyzed immortalized cell lines. The results confirmed that the cells from the OvCa3A hTERT cell line did not express the CD133 molecule, whereas the cells from OvCa7 A hTERT were strongly positive for the CD133 marker (Figure 4b and Figure S2). Both cell lines OvCa3 A hTERT and OvCa7 A hTERT expressed Pax8 (Figure 4c and Figure S3). The CD133 relative expression ratio was 0.22 for OvCa7 A hTERT cells and 0.32 for HEPC-CB.1 cells. On the other hand, the Pax8 relative expression ratio was 0.93 for OvCa3 A hTERT cells, 1.10 for OvCa7 A hTERT cells, and 1.57 for OAW-42 cells, which served as the positive control (Figure 4d). These results indicated a compatibility with the findings obtained from immunofluorescence staining.

### 2.2. Metabolic Activity and Migration Activity of Primary Ovarian Cancer Cells and Immortalized Ovarian Cancer Cells

**Figure 5:**
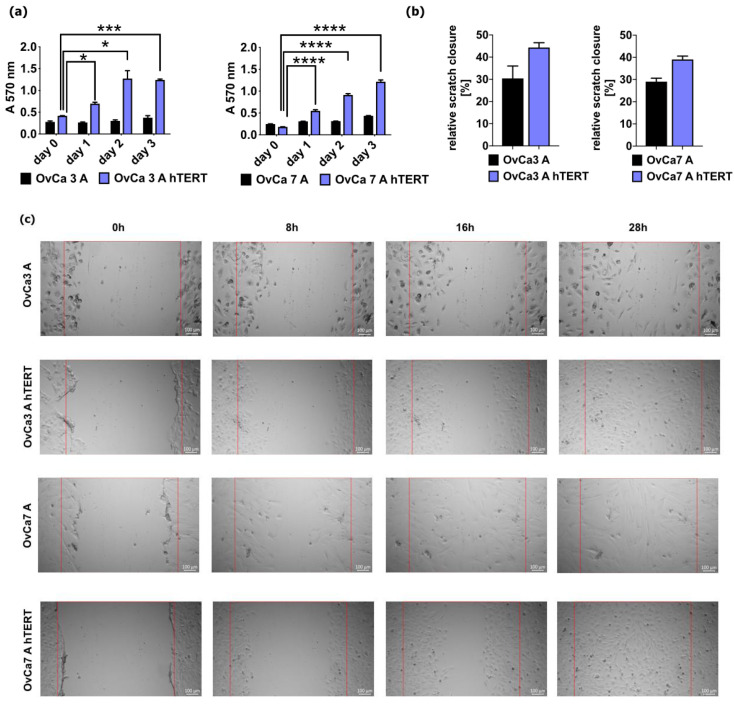
Metabolic activity and migration activity of primary ovarian cancer cells and immortalized ovarian cancer cells (**a**) The bar charts show absorbance at 570 nm for primary ovarian cancer cells and immortalized cancer cells at different time points. For the primary cells, the data represent mean ± SEM from experiments performed in triplicate; for immortalized cells, the data represent mean ± SEM values from three independent experiments performed in triplicate. **** *p* < 0.0001, *** *p* < 0.001, * *p* < 0.05 calculated vs. day 0 (**b**) The bar charts show relative wound closure for primary ovarian cancer cells and immortalized ovarian cancer cells. For the primary cells, the data represent mean ± SEM from experiments performed in duplicate, while for immortalized cells, the data represent mean ± SEM values from three independent experiments performed in duplicate. (**c**) Representative images of scratch closure at different time points. The top panel shows images for primary ovarian cancer cells OvCa3 A and immortalized ovarian cancer cells OvCa3 A hTERT, and the bottom panel shows images for primary ovarian cancer cells OvCa7 A and immortalized ovarian cancer cells OvCa7 A hTERT. The red lines represent scratch area. The scale bars represent 100 μm.

To determine the biological activity of primary ovarian cancer cells and immortalized ovarian cancer cells, the MTT assay and scratch test were performed. The metabolic activity of both immortalized ovarian cancer cell lines was higher compared to primary ovarian cancer cells. For the primary cells OvCa3 A and OvCa7 A, metabolic activity slowly increased and reached the highest value on day 3 (for OvCa3 A, 0.37 ± 0.01, and for OvCa7 A, 0.43 ± 0.01). For OvCa3 A hTERT, metabolic activity increased from day 1 (0.60 ± 0.03) to day 2 (1.27 ± 0.18) and was detectable at a similar level on day 3 (1.24 ± 0.02, *p* < 0.001 vs. day 0). For OvCa7 A hTERT, metabolic activity increased gradually from day 1 (0.30 ± 0.01) to day 3 and reached the highest value on day 3 (1.21 ± 0.01, *p* < 0.0001 vs. day 0) (Figure 5a).

As with metabolic activity, the migration activity of immortalized ovarian cancer cells was higher compared to the corresponding primary ovarian cancer cells. It was shown that relative wound closure was higher for the immortalized OvCa3 A hTERT cells compared to the primary OvCA3 A cells (44.3% ± 2.1% vs. 30.4% ± 5.5%, respectively) and for the OvCa7 A hTERT cells compared to the OvCA7 A cells (39.0% ± 1.5% vs. 29.0% ± 1.6%, respectively) (Figure 5b,c).

### 2.3. ALDH1 Activity in Primary and Immortalized Ovarian Cancer Cells

**Figure 6:**
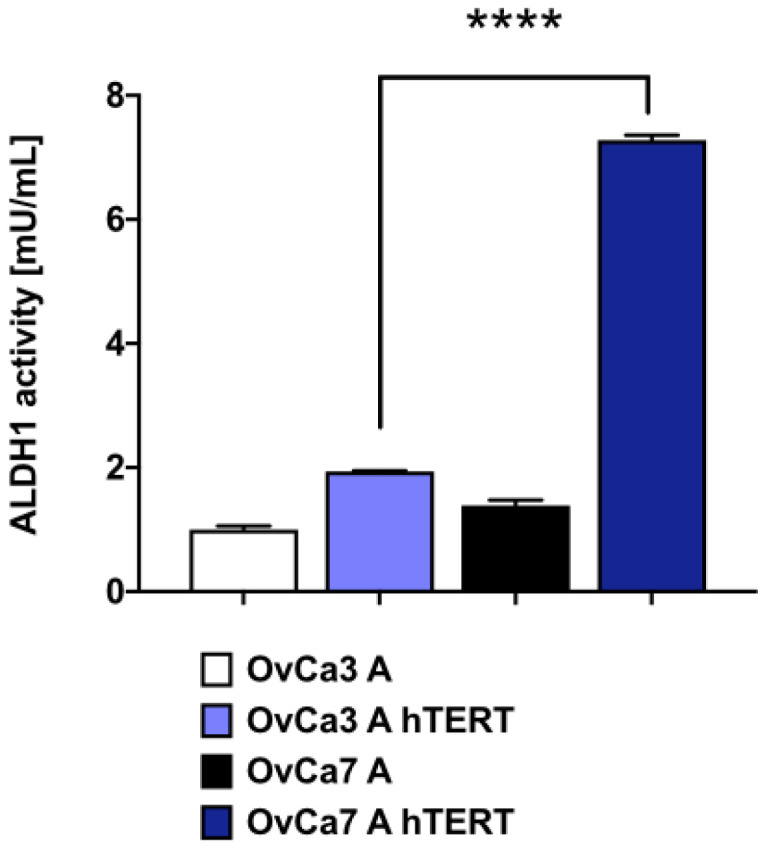
ALDH1 activity in immortalized ovarian cancer cells. For primary cells, the data represent mean ± SEM from duplicate, while for immortalized cells, the data represent mean ± SEM values from three independent experiments performed in duplicates. **** *p* < 0.0001 calculated vs. OvCa3 A hTERT.

To determine ALDH1 activity in primary and immortalized ovarian cancer cells, the colorimetric assay was performed. For primary ovarian cancer cells, the lower ALDH1 activity was observed compared to corresponding immortalized cells: for OvCa3 A (1.00 mU/mL ± 0.10 mU/mL vs. 1.92 mU/mL ± 0.01 mU/mL), and for OvCa7 A (1.33 mU/mL ± 0.10 mU/mL vs. 7.20 mU/mL ± 0.05 mU/mL), respectively. In addition, ALDH1 activity for the OvCa7 A hTERT cells was over three times higher than for the OvCa3 A hTERT cells (7.20 mU/mL ± 0.05 mU/mL vs. 1.92 mU/mL ± 0.01 mU/mL, respectively; *p* < 0.0001) (Figure 6).

### 2.4. Characteristics of Spheroids Derived from Primary and Immortalized Ovarian Cancer Cells

**Figure 7:**
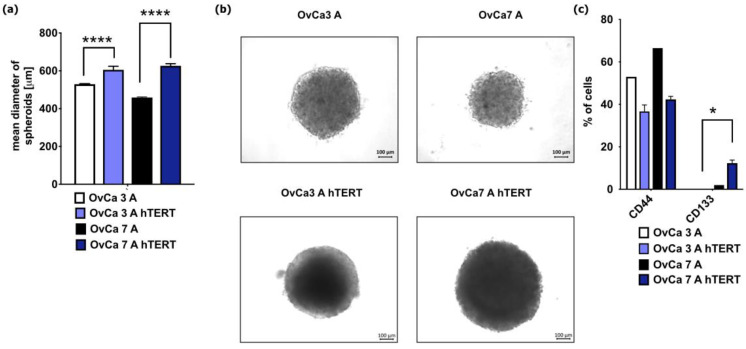
Characteristics of spheroids derived from immortalized ovarian cancer cells. (**a**) Mean diameter of spheroids (μm). The data represent mean ± SEM values from 10 spheroids. **** *p* < 0.0001 calculated vs. OvCa3 A hTERT or OvCa7 A hTERT. (**b**) Representative images of spheroids. The scale bars represent 100 μm. (**c**) The bar chart shows the percentage (%) of positive cells for selected markers (CD133, CD44). For primary cells, the data represent results from one experiment, while for immortalized cells, the data represent mean ± SEM values from three independent experiments. * *p* < 0.05 calculated vs. OvCa3 A hTERT.

Spheroids were formed to assess the ability of primary and immortalized cells to grow in 3D conditions. The average diameter of spheroids derived from primary cells OvCa3 A and OvCa7 A was lower compared to corresponding immortalized cell lines (OvCa3 A 529.3 μm ± 6.3 μm vs. OvCa3 A hTERT 605.2 μm ± 6.3 μm, *p* < 0.0001) (OvCa7 A 458.7 μm ± 6.3 μm vs. OvCa7 A hTERT 626.3 μm ± 3.7 μm, *p* < 0.0001) (Figure 7a,b).

To assess the presence of CSCs within the formed spheroids, the expression of selected markers characteristic for CSCs was determined using flow cytometry analysis after spheroid decomposition for single cells. For spheroids derived from primary OvCa3 A, there was no expression of CD133, while for spheroids from OvCa7 A, the small population of cells was positive for CD133 (2.00%). As previously shown in a 2D culture, OvCa3 A hTERT cells did not express CD133 in a 3D model, whereas for the OvCa7 A hTERT spheroids, the percentage of CD133-positive cells amounted to 12.33% ± 1.45% (*p* < 0.05). Similarly to cells in 2D model, the spheroids derived from OvCa3 A and OvCa7 A cells were positive for CD44 (53.00% vs. 66.50%, respectively). There were no differences in the expression of CD44 markers for cells derived from the OvCa3 A hTERT spheroids (36.66% ± 1.76% of the population) or the OvCa7 A hTERT spheroids (42.33% ± 1.45% of the population) (Figure 7c).

## 3. Discussion

Ovarian cancer of epithelial origin of the histological type of high-grade serous carcinoma is the most common malignant tumor in women with a fatal prognosis for survival due to a lack of early diagnosis and susceptibility for recurrence because of resistance to current chemotherapeutic protocols [22,23,24]. Extensive research on the ovarian cancer cell biology and targeted therapies focused on CSCs are necessary to develop effective treatment protocols. The advantage of using primary tumor cells from ascites or ovarian cancer tissue in CSC studies is that within heterogeneous tumor cell populations, there is a subpopulation representing CSCs responsible for tumor progression. However, there are still some difficulties, including a limited number of isolated cancer cells, short cell lifespan, and a small population of the CSCs in the heterogeneous material. In primary human cells, in which telomere-controlled senescence is the sole difficulty preventing unlimited lifespan, exogenous hTERT can be used to immortalize the cells, especially since almost 90% of cancers show overexpression of hTERT [14]. The post-operative tissue of ovarian cancer and the ascitic fluid are the main sources of primary cancer cells. Importantly, primary cancer cells can reflect the phenotype and genetic background of the tumor. However, the inter-patient heterogeneity of the cancer and the efficacy of cancer cell isolation is a great obstacle to experiment reproducibility when using samples from the primary solid tumor. Ascitic fluid is easily accessible and isolation of cancer cells is quite straightforward, but the number of cells may be insufficient for experiment repetitions and appropriate statistical analysis. Immortalization of cells using hTERT gives an opportunity to perform an appropriate number of repetitions due to the high proliferative activity of the cells. However, immortalization may affect the cell phenotype and their biological activity.

Cytomorphological analysis of primary cells from serous ovarian carcinoma before hTERT immortalization revealed four distinct subpopulations of cancer cells assessed according to the cytological criteria recommended by *Essentials of Fluid Cytology Atlas* [25]. The primary cellular composition of the ascitic fluid collected from patients with serous ovarian carcinoma showed similarities with the previously published reports, and the origin of ovarian epithelial cells was confirmed with the expression of CA 125 and CK8 [23]. After immortalization, only two cell types showed a growth advantage. The morphological characterization of the immortalized cells showed that two cell populations were established in both analyzed cases, with similar features to type 1 and 4 of primary cancer cells before immortalization. Type 1 of hTERT-immortalized cells presented typical malignant features, such as large size and round shape, central round and large nuclei, high nuclear density, and high nuclear-cytoplasmic ratio (2:1), and this subpopulation were more homogenous compared to the primary subpopulation of type 1. The second type of immortalized cells showed atypical morphological features comparable with type 4 of primary cells, with a distinct cell border and basophilic cytoplasm and inverse nuclear-cytoplasmic ratio (1:3) compared to malignant cells. These results suggest that only two cell subpopulations have the biological predispositions to growth. Based on published data, it can be assumed that only the cell clones with unique stemness features were selected during the immortalization procedure [26,27]. This suggestion might be confirmed by the high nuclear accumulation of the p53 protein found in the majority of immortalized cells, which indicated that only malignant cells were able to survive and proliferate [7]. Strong p53 immunopositivity in immortalized cells found in the current study indicates that the selection of cells carried the TP53 gene mutation, similar to the published data on the correlation between p53 overexpression and gene mutation [28,29,30].

Our previous study showed that the commercially available ovarian cancer cell lines ES-2 and OAW-42 expressed the markers characteristic for the mesenchymal stem cell markers CD73 (ecto-5′-nucleotidase), CD90 (Thy-1), and CD105 (endoglin) [31]. Expression of these markers in cancer cells is associated with EMT and poor prognosis of ovarian cancer patients’ survival. This study revealed that the immortalization affects the phenotype of the primary cells. For OvCa 3 A hTERT, an increased number of CD105-positive cells was observed compared to primary OvCa3 A cells, whereas for OvCa7 A hTERT, an increased number in CD90-positive cells and a decreased number in CD73- and CD105-positive cells was detected.

CD73 expression was observed on cancer-initiating cells. In high-grade serous ovarian cancer, the overexpression of the CD73 molecule with EMT markers (e.g., Snail, vimentin, or Twist1) was associated with poor prognosis of survival. Furthermore, it was shown that the expression of CD73 led to the escape of tumor cells from immune system control and induced tumor growth [24]. The silencing of CD73 expression via shRNA or using neutralizing antibodies decreased the formation of spheroids by the primary ovarian tumor cells and inhibited tumor growth in a mouse model. Moreover, the knockdown of the CD73 molecule caused a reverse from the mesenchymal to the epithelial transition phenotype of ovarian cancer cells [32]. The knockdown of the CD90 molecule in A2780 (ovarian cancer) cells decreased the proliferative activity of cells, relative cell growth (self-renewal potential), and expression of the pluripotency-related markers Nanog and Sox2. The ovarian cancer A2780 cell line, sorted for two populations with or without CD90 expression, revealed an increased proliferation activity and self-renewal in CD90-positive cells’ population. In addition to the in vitro experiments in the tissue section, the expression of CD90 RNA was observed with in situ hybridization. Overall, the high expression of CD90 was associated with poor prognosis of survival in high-grade serous ovarian cancer and endometroid ovarian cancer [33]. The CD105 molecule is involved in ovarian cancer metastasis through the activation of EMT via the inhibition of NDRG1, E-cadherin [34]. The overexpression of CD105 with the co-expression of CD44 and CD106 was determined in paclitaxel-resistant ovarian cancer cell lines and primary ovarian cancer cells resistant to different chemotherapeutics. This phenotype of cells correlated with the shorter survival of ovarian cancer patients, the presence of metastasis to different organs, and fast cancer relapse [35].

In our study, the presence of the pluripotency-related markers *Oct4*, *Sox2*, and *Nanog* and the protooncogenic markers *p53*, *p21*, and *c-myc* in primary ovarian cancer cells and immortalized ovarian cancer cells was confirmed using the RT PCR and/or immunofluorescence staining. For OvCa3 A hTERT, only *Oct4* was upregulated, whereas *Sox2*, *p53*, *p21*, and *c-myc* were downregulated. Different results were obtained for OvCa 7 A hTERT, in which *Sox2* and *p53* were upregulated, and *Oct4*, *p21*, and *c-myc* were downregulated. Wang et al. showed that the immortalization of MSCs using hTERT increased the expression of the pluripotency-related markers Sox, Nanog, and Klf4 and protooncogenic myc and TP53 compared to primary cells [36]. A study conducted by Li et al. showed no significant changes in the expression of p21 in immortalized human ovarian surface epithelial cells compared to primary cells. Moreover, the immortalization did not affect the function of the p53 and pRb protein [37]. A study conducted by Samardzija et al. [38] showed that the expression of Oct4 analyzed using IHC staining was higher in the tissue samples of an increased pathological grade of serous ovarian cancer. Furthermore, the expression of Oct4 analyzed using RT-PCR was higher in cells isolated from the ascitic fluid of patients with recurrent epithelial ovarian cancer compared to patients not treated with chemotherapy. The silencing of *Oct4* in the Hey cell lines (an invasive ovarian cancer cell line) using shRNA leads to lower proliferation activity and migration ability. Moreover, the cells became more sensitive to cisplatin. In a mouse model, an intraperitoneal injection of the knockdown Hey cells caused a reduction in tumor size and tumor aggressiveness that led to a prolonged lifespan of mice compared to Hey cells with the expression of *Oct4* [38]. A study performed by Wang et al. [39] showed that the Src kinase, which is a target of Sox2, is responsible for the different features of serous ovarian cancer cells, such as migratory activity, adhesion properties, and invasiveness. The silencing of Src kinase leads to a decreased migration in Ho8910 ovarian cancer cells and increases the adhesion of cells to different extracellular matrix proteins, such as laminin or collagen I [39]. Bareiss et al. documented that Sox2-expressing cells are more resistant to cell death the via apoptotic pathway caused by different chemotherapeutic agents or TRAIL [40]. Yun et al. [41] demonstrated that the silencing of Nanog by siRNA caused a decreased migration activity and invasiveness of two analyzed human ovarian cancer cell lines, SKOV-3 and A2780. Furthermore, the silencing of *Nanog* decreased the expression of EMT markers, such as vimentin, N-cadherin, and ZEB1, and the activation of the AMPK/mTOR signaling pathway. Overall, the results suggest that the high expression of *Nanog* and low expression of pAMPK (phosphorylated AMPK) is associated with poor prognosis of patient survival and resistance to platinum-based chemotherapy. *C-myc* is an important factor involved in different pathways, e.g., cell survival, progression of the cell cycle, and proliferation activity [42]. Moreover, *c-myc* regulates the expression of vimentin. In different types of tumors, the overexpression of *c-myc* was related to resistance to different chemotherapeutics [42]. The silencing of *c-myc* by siRNA in cisplatin-resistant A2780 cells leads to decreased cell proliferation, survival, and inhibition of cell cycle progression [42]. p53 is a tumor suppressor protein involved in the activation of proteins that play a crucial role in the repair of DNA after different types of damage. Moreover, p53 arrested the cells at the G1 checkpoint and induced apoptosis when the repair of DNA was impossible [43]. In ovarian cancer, the mutation of p53 occurs in 90% of epithelial cancers. The mutation of p53 can be used as a screening marker and prognostic marker of epithelial ovarian cancer. Furthermore, a molecule targeting the mutated p53, named PRIMA-1MET, combined with carboplatin chemotherapy is used in the II phase of clinical trials of recurrent high-grade serous ovarian cancer (NCT02098343). p21 is known as an inhibitor of cell cycle progression and proliferation [44]. The tissue section of ovarian cancer showed an overexpression of p21 [45]. The silencing of cytoplasmic p21 in cisplatin-resistant ovarian cancer cell line C13* decreased cell survival. Moreover, the inhibition of the translation of p21 into cytoplasm reduced resistance to cisplatin, in contrast to the translocation of p21 into cytoplasm. Cytoplasmic p21 may be a potential biomarker of cisplatin-resistant ovarian cancer [46]. Overall, pluripotency and proto-oncogenic markers may constitute biomarkers and potential targets for ovarian cancer treatment.

Studies on ovarian tumors also employed the expression of transcriptional factor Pax8, involved in the development of the fallopian tube epithelium and the development of Mullerian phenotype cancers (serous, clear cells, and endometroid) [47]. Pax8 is expressed in almost 90% of high-grade serous ovarian cancer and is a commonly used marker to classify ovarian serous tumors [48]. Both immortalized cell lines, OvCa3 A hTERT and OvCa7 A hTERT, express Pax8, which confirmed their epithelial origin, and is in line with similar pattern reported for OVPA8 cell line established after numerous passages [8].

EMT is a crucial process associated with cancer invasiveness and metastasis. In this process, cancer cells change their phenotype from epithelial to mesenchymal [49]. In the present study, the presence of Snail and vimentin in primary and immortalized ovarian cancer cells was detected using immunofluorescence staining. Snail, Slug, and vimentin were upregulated in cisplatin-resistant A2780 cell lines, as determined via genomic and proteomic analysis. The knockdown of Snail and Slug led to a reverse mesenchymal phenotype compared to the epithelial phenotype of cisplatin-resistant A2780 cells [50]. Vimentin, a protein of intermediate filaments, is the main important factor in the EMT marker. During EMT, cancer cells become elongated, which is associated with changes in cytoskeleton organization mainly via vimentin. Vimentin also affects tumor angiogenesis via the activation of the NOTCH signaling pathway [51].

The CSC phenotype was assessed for primary and immortalized ovarian cancer cells based on the expression of CD133, CD24, and c-kit molecules. In the research on CSCs, CD133 is considered as a marker for the identification of CSC populations in different solid tumors, including ovarian cancer [17]. Importantly, for the immortalized OvCa7 A hTERT, an increase was observed in the percentage of CD133-positive cells to around 90%. Furthermore, the overexpression of the CD133 molecule in OvCa7 A hTERT was confirmed using the RT-PCR and Western blot. Due to the collection of the negative population for the CD133 molecule after the magnetic-activated cell sorting of OvCa3 A cells, both primary OvCa3 A and OvCa3 A hTERT were negative for CD133. The OvCa7 A cells were positive for CD24; however, the expression of CD24 was not detected after immortalization. This observation suggests that only a specific clone of the OvCa7 A cells expressing CD133 was immortalized. C-kit was detected in primary and immortalized ovarian cancer cells. Consequently, the OvCa7 A hTERT cell line may be used in further research to mimic CSC features in terms of CD133 expression, in contrast to OvCa3 A hTERT. A study conducted by Zhang et al. observed a high expression of CD133 in patients with high-grade serous ovarian cancer, diagnosed in late stages with poor prognosis of prolonged survival. Moreover, the patients did not respond to chemotherapeutic agents [52]. The spheroids derived from the SKOV-3 cells increased tumor formation and growth in a mouse model. Importantly, in the tissue section, a high expression of CD44, CD133, Ki67, and the pluripotency-related markers Oct4 and Nanog was detected. This was associated with the downregulation of the genes involved in cell adhesion and the upregulation of the genes important for the survival of cells and cell cycle progression [53]. CD24 affects the EMT via the activation of the Akt- and ERK-signaling pathways in the cisplatin-resistant ovarian cancer cell line Caov-3. The activation of EMT leads to the increased proliferation and metastatic potential of cells. Moreover, CD24 expression was observed in ovarian cancer tissue sections, and was associated with metastasis into distant lymph nodes and the peritoneal cavity [54]. A study conducted by Chau et al. [55] showed that the postulated mechanism of c-kit action is activation of the Wnt/β-catenin signaling pathway and the ATP-binding cassette transporter G2. Furthermore, a hypoxic condition can enhance the expression of the c-kit marker [55]. The silencing of the c-kit marker via siRNA leads to a decreased number of ovarian tumor-initiating cells, tumorigenic potential, and expression of CSC markers. Importantly, a clinically applied inhibitor of c-kit kinase (an Imatinib brand called Gleevec) also decreased the number of ovarian tumor-initiating cells. In our study, primary and immortalized cells were determined for the activity of ALDH1, and the ALDH1 level was found to be three times higher in the obtained CD133-positive cell line OvCa7 A hTERT compared to the CD133-negative cell line OvCa3 A hTERT. For both primary cells OvCa3 A and OvCa7A, the ALDH1 activity was lower compared to corresponding immortalized cell lines. Studies by Choi et al. revealed that double positive ALDH1 (+)/CD133 (+) ovarian cancer cells, which are highly resistant for chemotherapy, could generate a heterogeneous population of cancer cells [56]. ALDH1 activity is one of the most important features not only in normal stem cells, but also in CSCs. ALDH1 directly protects the cells from toxic aldehydes and indirectly from reactive oxygen species (ROS) [57]. The expression of ALDH1 in patients with high-grade serous ovarian cancer correlated with the resistance to platinum-based chemotherapy and poor prognosis [58]. A study performed by Kuroda [59] showed that cells with high expression of ALDH1 could be isolated from different ovarian cancer cell lines (serous ovarian cancer and clear cell carcinoma). Cells with a high expression of ALDH1 were more tumorigenic and aggressive, as shown in a mouse model [59].

Our results showed higher metabolic and migration activity of the two analyzed immortalized ovarian cancer cells OvCa3 A hTERT and OvCa7 A hTERT compared to the corresponding primary cells. The higher metabolic activity may have been related to higher proliferation activity. A study conducted by Wang et al. [36] showed that the immortalization of MSCs using hTERT increased proliferation ability compared to primary cells. Telomerase activity is a potential biomarker of cell proliferation. The hTERT affects cell proliferation not only by preventing telomeres from shortening, but also by activating the epidermal growth factor receptor (EGFR) [60]. Research on telomerase activity in the nasopharyngeal carcinoma radioresistant cell line CNE-2R documented that the expression of stem cell-related genes (including CD133 mRNA) and the hTERT gene in radioresistant CNE-2R cells was higher than those in radiosensitive CNE-2 cells. The radioresistant cell line CNE-2R showed cancer stem cell-like characteristics (Oct4, Sox2, Nanog, Bmi1, and CD133), especially in the sorted cells that were CNE2R-CD133-positive, and, in contrast to the CNE-2R-CD133-negative cells, revealed higher proliferation activity, tumorigenesis capacity, and telomerase activity [61]. RNA sequencing performed by Liu et al. revealed that the transfection of the osteosarcoma cells U2OS and the immortalized fibroblast cells VA-13 with plasmids carrying out hTERT and mutated hTERT increased the expression of a gene involved in cell adhesion. This was also confirmed in an in vitro assay, in which the transfected cells showed increased adhesion to the ECM protein fibronectin. Moreover, increased migration and transmigration activity of U2OS cells was observed [62]. On the other hand, the silencing of hTERT via siRNA in esophageal squamous carcinoma cells decreased cell migration and invasiveness, but did not affect cell proliferation [63].

Our study revealed no differences in the size of the spheroids formed from two immortalized ovarian cancer cell lines. However, the size of the spheroids derived from the immortalized ovarian cancer cell lines was greater than that of the spheroids from primary ovarian cancer, as presented in the current and our recent study [64]. Nevertheless, differences were observed in the spheroid phenotype. For spheroids derived from OvCa3 A, as expected, there was no expression of CD133, while for OvCa7 A spheroids, the small population of cells was positive for CD133. In spheroids of OvCa7 A hTERT, a significantly higher percentage of cells expressed the CSC phenotype CD133, whereas in spheroids from OvCa3 A hTERT, CD133 cells were not detectable.

## 4. Materials and Methods

### 4.1. Isolation of Human Primary Ovarian Cancer Cells

Primary ovarian cancer cells were isolated from the ascitic fluid aspirated from two patients with permission from the Bioethics Committee at the Medical University of Wroclaw (No. KB-489/2020). Both patients suffered from high-grade serous ovarian cancer. Medical procedures were carried out at the Department of Gynecology and Obstetrics, Wroclaw Medical University in Poland. The cells were isolated from the ascitic fluid, as previously described [64]. The obtained ascitic fluid was centrifuged for 10 min 1000× *g*. The cells were washed in PBS twice. Finally, the isolated cells were resuspended in a culture medium (DMEM, Thermo Fisher Scientific, Carlsbad, CA, USA) containing 10% FBS (Thermo Fisher Scientific, Carlsbad, CA, USA), a 1% penicillin/streptomycin solution (Thermo Fisher Scientific, Carlsbad, CA, USA), and 1% L-glutamine (Thermo Fisher Scientific, Carlsbad, CA, USA), and cultured in standard conditions (37 °C, 5% CO_2_).

### 4.2. Isolation of CD133 Positive and Negative Cells-Magnetic-Activated Cell Sorting

The cells derived from the ascitic fluid of patients with high-grade serous ovarian cancer were magnetically sorted using CD133 beads (Milteni Biotec, Bergisch Gladbach, Germany) and an MS column (Milteni Biotec, Bergisch Gladbach, Germany) according to the manufacturer’s instructions. In total, 1 × 10^6^ cells were centrifuged at 300× *g* for 10 min. After centrifugation, the cell pellet was resuspended in 40 μL of a sorting buffer (PBS with 2% BSA and 2 mM EDTA, pH = 7.2, IIET, Wroclaw, Poland). Next, 10 μL of the anti-human CD133-Biotinyled antibody was added. The cells were incubated on ice for 15 min. After incubation, the cells were washed with a sorting buffer and centrifuged at 300× *g* for 10 min. The cell pellet was resuspended in a sorting buffer with the addition of 20 μL of Anti-Biotin MicroBeads and incubated on ice for 15 min. Next, the cells were washed with a sorting buffer and centrifuged at 300× *g* for 10 min. After centrifugation, the cell pellet was resuspended in 500 μL of a sorting buffer. Before magnetic separation, the MS column was placed in a magnetic field and activated with a sorting buffer. The cell suspension was then applied to the MS column. The flowthrough contained unlabeled cells representing a CD133-negative cell population. Subsequently, the column was washed with an appropriate volume of a sorting buffer, and the flowthrough from this step was combined with unlabeled cells. A CD133-positive population was obtained when the column was removed from the magnetic field and washed with a sorting buffer. In the final step, the negative and positive populations of isolated cells were centrifuged at 300× *g* for 10 min. Next, the cells were resuspended in a culture medium (DMEM) containing 10% FBS (Thermo Fisher Scientific, Carlsbad, CA, USA), a 1% penicillin/streptomycin solution (Thermo Fisher Scientific, Carlsbad, CA, USA), and 1% L-glutamine (Thermo Fisher Scientific, Carlsbad, CA, USA) and cultured in standard conditions (37 °C, 5% CO_2_). Because the number of obtained CD133-positive cells was insufficient, the cells were not able to proliferate and could not be used in further experiments.

### 4.3. Immortalization of Primary Ovarian Cancer Cells

Initially, the cells derived from the patients’ ascitic fluid presented a highly heterogeneous population of various cell types including cancer cells, mesothelial cells, and fibroblast-like cells. To remove fibroblasts and mesothelial cells from the primary culture, cells were detached from plastic surface after 2 min of exposure to trypsin. Cells showing a fibroblast-like morphology, but not epithelial cancer cells, were removed, and the culture medium supplemented with FBS was added to inactivate the remaining trypsin. The transfection was performed on cells with significantly higher adhesion to plastic surface with cancerous cell morphology and clustered growth. The primary ovarian cancer cells were transfected with the hTERT plasmid (pBABE-puro-hTERT, www.addgene.org) after second passage and selected with Puromycin (Merck, Kenilworth, NJ, USA). The ViaFect™ Transfection Reagent (Promega, Mannheim, Germany) was applied for transfection according to the manufacturer’s protocol. A day before the transfection, the cells were seeded in a 24-well plate (2.0 × 10^4^ cells/well) in the OptiMEM medium supplemented with GlutaMAX (Thermo Fisher Scientific, Carlsbad, CA, USA) and 3% of FBS (Thermo Fisher Scientific, Carlsbad, CA, USA). Before the transfection, the culture medium with FBS was replaced with a serum-free Opti-MEM medium. Two micrograms of DNA plasmids were mixed with 200 μL of the serum-free Opti-MEM medium and 6 μL of the ViaFect™ transfection reagent. Following 20 min of incubation at room temperature (RT), the DNA-Transfection Reagent complex was mixed with 2 mL of Opti-MEM medium, and 0.5 mL of the mixture was applied to each well of a 24-well plate. After 24 h of incubation at 37 °C, 5% CO_2_, 0.5 mL of Opti-MEM medium with 3% of FBS was applied to each well and exchanged after 48 h. All transfection details are presented in our previous paper [65]. The immortalized cell lines were called OvCa3 A hTERT and OvCa7 A hTERT, as they were isolated from the ascitic fluid of patients with ovarian cancer numbers 3 and 7, respectively.

### 4.4. Characteristics of Primary Ovarian Cancer Cells and Immortalized Ovarian Cancer Cells

The primary cells were used for all experiments from passage 1 to passage 3, while immortalized cells were used for all experiments from passage 1 to passage 5.

#### 4.4.1. Cytological Assessment

For cytological assessment, primary cells from passage 2 and immortalized cells from passage 2 were used. For cytospin preparation, the cells were seeded at a density of 5 × 10^3^ cells/100 µL PBS per drop on slides. The slides were kept in 37 °C for 30 min to increase cell adhesion. Next, the specimens were fixed in cold acetone for 15 min, stained with hematoxylin and eosin, and were examined cytologically according to the standard protocol [25]. The cytospin specimens were assessed with a 40× dry objective of an Olympus microscope (BX61, Olympus, Japan) and Cell Sens Dimension version 3.2 Image Acquisition, Process Analysis Software documentation.

#### 4.4.2. Immunocytochemical Staining (ICC)

To assess the cancerous feature of cells isolated from ascitic fluid the immunohistochemical staining for the presence of the p53 protein, CA 125 antigen and cytokeratin 8 (CK8) was performed on cytospin specimens using and the Universal Dako REAL EnVision Detection System, Peroxidase/DAB+, Rabbit/Mouse (Dako, Copenhagen, Denmark). Endogenous peroxidase reactivity was blocked with the Dako REAL Peroxidase Blocking Solution (Dako, Copenhagen, Denmark). Next, the cytospin specimens were incubated with primary antibodies (Table S1) for 60 min at RT. After washing, with 0.1 M Tris buffer, pH = 7.4 (TBS), cells were incubated with Dako REAL EnVision/HRP, Rabbit/Mouse (Dako, Copenhagen, Denmark) for 30 min at RT. The binding of the antibody was visualized using DAB (3,3 diaminobenzidine) chromogen (Dako, Copenhagen, Denmark) for four minutes at RT. The sections were counterstained with hematoxylin and mounted. The TBS buffer without primary antibodies was used as a negative control. Positive controls for each antibody were performed according to the manufacturer’s recommendation. The following positive controls were used: CA125 antigen-ovarian serous adenocarcinoma tissue from patient with high serum level of CA125 antigen, cytokeratin 8 (CK8)-ovarian serous adenocarcinoma tissue, and p53 antigen-OvBH-1 cell line [7,18].

The expression of the analyzed proteins on cell cytospines was assessed semi-quantitatively, taking into account the intensity staining and the number of cells showing immunoreactivity for the p53 protein, CA 125, and CK8. The percentage of immunopositive cells was determined by counting 1000 cells in randomly selected areas using an Olympus BX51 microscope (Olympus, Tokyo, Japan). The cell specimens were assessed by cytopathologist J.B. twice, and the average value from two independent analyses was taken into account. Staining was scored as positive when more than 5% of cells showed immunoreactivity. The intensity score was based on the color of the reaction and was assessed as follows: 0 = no immunostaining, light yellow color = weak (+), medium brown color = moderate (++), and strong brown color = strong (+++).

#### 4.4.3. Flow Cytometry Analysis

The primary ovarian cancer cells (passages 1–3) and immortalized ovarian cancer cells (passages 1–3) were analyzed using flow cytometry. After trypsinization (IIET, Wroclaw, Poland), the cells were washed with PBS and stained with the fluorochrome-conjugated anti-human PE-conjugated antibodies CD73, CD90, CD105, CD44, CD45, CD133 (BD Biosciences, San Jose, CA, USA), FITC-conjugated CD24, CD34 antibodies (BD Biosciences, San Jose, CA, USA), and isotype controls (BD Biosciences, San Jose, CA, USA) at 4 °C for 30 min. The cells were then washed with PBS (IIET, Wroclaw, Poland) and analyzed using a BD LSRFortessa flow cytometer (BD Biosciences, San Jose, CA, USA). The data were processed with Flowing Software 2 version 2.5 (Perttu Terho, Turku Centre for Biotechnology, Turku, Finland), and appropriate histograms were prepared.

#### 4.4.4. Immunofluorescence Staining and Microscopic Imaging

The primary ovarian cancer cells (passage 2) and immortalized ovarian cancer cells (passage 2) were stained for the expression of selected markers: p53, Pax8, CD133, PDGFRα, CD44, FAP, Snail, Oct, Sox, Nanog, vimentin, c-kit, and F-actin. Before immunostaining, the cells were fixated with 4% PFA for 15 min at RT. Next, to analyze the expression of markers with nuclear localization, the cell membrane was permeabilized with 0.1% Triton X100 (Avantor Performance Materials Poland, Gliwice, Poland) for 15 min at RT. After permeabilization, the unspecific binding of antibodies was blocked with 1% BSA (Symbios, Gdansk, Poland) for 40 min at RT. Subsequently, the cells with selected primary antibodies were incubated for 1 h at RT or overnight at 4 °C (Table S1). The cells were stained with secondary antibodies Alexa Fluor 488-conjugated goat anti-mouse IgG and/or Alexa Fluor 647-conjugated goat anti-rabbit IgG (Invitrogen, Carlsbad, CA, USA) for 45 min (RT). F-actin was stained with Alexa Fluor 488-conjugated phalloidin (Invitrogen, CA, California, USA) for 45 min (RT) (Table S2). The NucBlue™ Fixed Cell ReadyProbes™ Reagent (DAPI) (Invitrogen, Carlsbad, CA, USA) was used to stain the cell nuclei for 30 min (RT). Images were acquired with a 40x dry objective of an Axio Imager Z2 microscope (Zeiss, Gottingen, Germany) and Zen Blue 2.6 Software (Zeiss, Gottingen, Germany). This software was also used to process the obtained images.

#### 4.4.5. Western Blot

Immortalized ovarian cancer cells (passage 3) were lysed in the RIPA Lysis and Extraction Buffer (Thermo Fisher Scientific, Carlsbad, CA, USA) and stored at −80 °C. In the next step, the total protein amount in the lysed samples was calculated using the BCA Protein Assay Kits (Thermo Fisher Scientific, Carlsbad, CA, USA). Each time, 50 μg of total protein was applied per line on SDS-PAGE gels. The WB analysis was performed according to the previously described method [66]. The cell extracts were loaded on SDS-PAGE and, after electrophoresis, were transferred onto an Immobilon PVDF Membrane (Merc, Kenilworth, NJ, USA). After semi-dry transfer, the membrane was blocked with a 5% solution of BLOT-QuickBlocker™ (Merc, Kenilworth, NJ, USA) for 1 h at RT. In the next step, an overnight incubation with primary antibodies against human CD133 (2F8C5 Mouse mAb, Invitrogen, Carlsbad, CA, USA), Pax8 (D2S2I Rabbit mAb, Cell Signaling Technology, Danvers, MA, USA), and β-Actin (13E5 Rabbit mAb, Cell Signaling Technology, Danvers, MA, USA) was applied at 4 °C. After washing three times with a 0.05% (*v*/*v*) Tween-20 (Thermo Fisher Scientific, Carlsbad, CA, USA) solution in PBS, the membrane was incubated with a secondary biotinylated antibody against rabbit (for β-Actin and Pax8) and mouse (for CD133) (Thermo Fisher Scientific, Carlsbad, CA, USA) for 1 h at RT. After the next washing in a 0.05% Tween-20/PBS solution, the membrane was incubated with streptavidin-HRP (Dako, Glostrup, Denmark) at RT for 1 h. The chemiluminescent reaction was developed using the ECL Western Blotting Substrate (Thermo Fisher Scientific, Carlsbad, CA, USA) and visualized on a CL-XPosure film (Thermo Fisher Scientific, Carlsbad, CA, USA). As a positive control, human HEPC-CB.1 cell lysate was used as the reference cell line positive for the CD133 molecule [67]. OAW-42 cell lysate was used as the reference cell line positive for Pax8, while Daudi cell lysate was used as the reference negative for Pax8. The densitometry analysis of CD133 and Pax8 was conducted using ImageJ software version 1.54f (National Institutes of Health, Bethesda, MD, USA). The results were presented as the ratio of CD133 and Pax8 protein expression levels to β-actin, which served as an internal control for normalization.

#### 4.4.6. Real-Time RT-PCR

RNA from the primary ovarian cancer cells (passage 2) and immortalized ovarian cancer cells (passage 3) was isolated using the NucleoSpin^®^ RNA Kit (Macherey-Nagel, Düren, Germany). The obtained RNA was then reverse-transcripted using the RevertAid First Strand cDNA Synthesis Kit (Thermo Fisher Scientific, Carlsbad, CA, USA). The expression of *Oct4*, *Sox2*, *p53*, *p21*, *c-myc*, and *CD133* was determined using appropriate TaqMan probes (Thermo Fisher Scientific, Carlsbad, CA, USA) and the TaqMan Master Mix (Thermo Fisher Scientific, Carlsbad, CA, USA). The reaction was performed with the ViiA 7 Real-Time PCR System (Applied Biosystems, Foster City, CA, USA). The housekeeping gene for calibration of the gene expression levels was GADPH. The results were analyzed using the ^ΔΔ^Ct method [64]. The primary ovarian cancer OvCa3 A served as a control for OvCa3 A hTERT, while OvCa7 A served as a control for OvCa7 A hTERT.

### 4.5. Metabolic Activity of Primary Ovarian Cancer Cells and Immortalized Ovarian Cancer Cells

An MTT assay was used to compare the metabolic activity of the primary ovarian cancer cells (passage 2) and immortalized ovarian cancer cells (passage 2 and 3). In total, 3 × 10^3^ cells were seeded at each well in 96-well plates. Metabolic activity was analyzed at four time-points (0 days, 1 day, 2 days, and 3 days). At each time-point, 4mg/mL of the MTT solution (Merc, Kenilworth, NJ, USA) were added to cells. The metabolic active cells converted the MTT reagent into formazan dye. The formazan dye was then dissolved in DMSO (Avantor Performance Materials Poland, Gliwice, Poland), and absorbance was read at 570 nm using a Wallac 1420 Victor2 Microplate Reader (Perkin Elmer, Waltham, MA, USA).

### 4.6. Migration Activity of Primary Ovarian Cancer Cells and Immortalized Ovarian Cancer Cells

The scratch test was used to compare the migration activity of primary ovarian cancer cells (passage 2) and immortalized ovarian cancer cells (passage 3). The cells were seeded onto 48-well plates at a density of 5 × 10^4^ per well. When cells fully covered the surface of the well, the scratch with 200 µL tips was made. The migration of cells was analyzed for 48 h with an interval of 4 h using an Axio Observer Microscope (Zeiss, Gottingen, Germany) equipped with a chamber for cell incubation (PeCon GmbH, Erbach, Germany). The cells were recorded at 37 °C 5% CO_2_ using a 5x dry objective (Zeiss, Gottingen, Germany). The video was acquired with Zen 2.6 Blue Edition Software (Zeiss, Gottingen, Germany). The same software was utilized for both video and image processing, as well as for measuring the scratch area in square micrometers (μm^2^) at both 0 h and 28 h post-scratch. The closure of the scratch area was determined by calculating the difference in the area between 0 h (A0h) and 28 h (A28h), with a total image size of 4,764,685 μm^2^. To obtain the relative scratch closure percentage, the scratch closure area in μm^2^ was multiplied by 100 and then divided by the total image size in μm^2^. This methodology for calculating relative scratch closure was consistent with previous descriptions, utilizing the area measurements in μm^2^ instead of pixels [68].

### 4.7. ALDH1 Activity in Primary and Immortalized Ovarian Cancer Cells

To determine the ALDH1 activity in primary (passage 3) and immortalized ovarian cancer cells (passages 4 and 5), the Aldehyde Dehydrogenase Activity Colorimetric Assay Kit (Merc, Kenilworth, NJ, USA) was used according to the manufacturer’s instructions. In total, 1 × 10^6^ cells were resuspended in 200 µL of ice-cold ALDH Assay Buffer and centrifuged at 13,000× *g* for 10 min. Next, the 50 µL of reaction mixes (including Sample and Standards of NADH in an ALDH Assay Buffer, ALDH Substrate Mix, and Acetaldehyde) were prepared and added into a 96-well plate. The plate was then incubated for 5 min at RT while protected from light. The initial absorbance at 450 nm was measured using a Wallac 1420 Victor2 Microplate Reader (Perkin Elmer, Waltham, MA, USA). The measurement of absorbance continued until the value of the most active sample was higher than the value of the highest standard. The activity of ALDH1 (mU/mL) was calculated according to the formulas from the Aldehyde Dehydrogenase Activity Colorimetric Assay Kit (Merc, Kenilworth, NJ, USA) guidelines.

### 4.8. 3D Cell Culture

Spheroids were formed at a dedicated 96-well plate Nunclon (Thermo Fisher Scientific, Carlsbad, CA, USA). At 96-well plates, the primary (passage 3) and immortalized ovarian cancer cells (passages 4 and 5) were seeded at a density of 1 × 10^4^ cells per well. The spheroids were formed for 2 days. Microscopic imaging of spheroids was performed using an Axio Observer Microscope (Zeiss, Gottingen, Germany) equipped with dry 10 × objectives. Images were acquired with the Zen 2.6 Blue Edition Software (Zeiss, Gottingen, Germany). This software was also used for image processing and calculation of spheroid diameters. Moreover, the spheroids were used for flow cytometric analysis for the expression of CD133 and CD44 markers. The spheroids were decomposed into single cells in a solution of the Accutase Cell Detachment Solution (Corning, Manassas, VA, USA) and shaken in a water bath (Thermo Fisher Scientific, Carlsbad, CA, USA) at 37 °C for 10 min. Subsequently, the cells were washed with PBS and stained with PE-conjugated anti-human antibodies CD44, CD133 (BD Biosciences, San Jose, CA, USA), and with the isotype controls (BD Biosciences, San Jose, CA, USA) at 4 °C for 30 min. The cells were then washed with PBS (IIET, Wroclaw, Poland) and analyzed using a BD LSRFortessa flow cytometer (BD Biosciences, San Jose, CA, USA). The data were processed with Flowing Software 2 version 2.5 (Perttu Terho, Turku Centre for Biotechnology, Turku, Finland), and appropriate histograms were prepared.

### 4.9. Statistical Analysis

Data analysis and preparation of graphs were performed using the GraphPad Prism version 7 (GraphPad Software Inc., San Diego, CA, USA). Statistical analysis was performed using a *t*-test.

## 5. Conclusions

In conclusion, we showed that primary ovarian cancer cells from the ascitic fluid may be successfully immortalized using plasmids carrying the hTERT gene. hTERT-immortalized ovarian cancer cells created in this study offer the advantage of being able to explore the “normal” characteristics of primary human cancer cells without the limitation of their lifespan. The obtained cells should be particularly useful to the study of the functional processes that are normally different during cancer development, such as cell cycle controls, adherence, motility, and regulation of gene expression, as well as their response to therapeutic strategies. This study shows that the immortalized ovarian cancer cells had higher proliferation activity than primary ovarian cancer cells. In general, both immortalized ovarian cancer cell lines reflected the phenotype of primary cancer cells, albeit with modifications. OvCa3 A hTERT kept the mesenchymal stem cell phenotype CD73+, CD90+, and CD105+, and was CD133-negative, whereas the cell population of OvCa7 A hTERT lost CD73 expression, but a majority of cells (almost 90%) expressed the CD133 characteristic for the CSCs phenotype. Furthermore, immortalized cells differed in gene expression level with respect to *Sox2* and *Oct4*, which was associated with stemness properties. The newly established unique immortalized cell line OvCa7 A hTERT with a characteristic of serous ovarian cancer malignancy feature, with the accumulation of the p53 and Pax8, and the overexpression of the CD133 and CD44 molecules, may be a useful tool in preclinical research on therapeutic approaches, especially those targeting the CSCs of ovarian cancer.

## Figures and Tables

**Table 1:** Cytomorphological features of cells from serous ovarian carcinoma before and after hTERT immortalization.

Cell Characteristics	Type of Ovarian Cell Subpopulation					
	Primary Culture				After Immortalization *	
	Type 1	Type 2	Type 3	Type 4	Type 1	Type 4
**Cell**						
Size	Large	Large	Large	Moderate	Large	Moderate
Shape	Round	Round	Round	Oval	Round	Oval
Borders	Indistinct	Indistinct	Distinct	Distinct	Distinct	Distinct
**Nucleus**						
Shape	Round	Round	Round	Oval	Round	Round
Localization	Central	Central	Central/atopic	Atypical	Central	Central
Hypernucleosis	+	+	−	−	+	−
Heteronucleosis	+	+	+	+	+	+
Hyperchromasia	+	−	−	−	+	+
Polynucleosis	−	−	−	−	−	−
Nucleoli	−	+	−	−	−/+	−
**Cytoplasm**						
Size	Moderate	Moderate	Large	Moderate	Moderate	Moderate
Staining	E	E	E	B	B	B
Vacuoles	−	single/numerous	numerous	−	−	−
**N/C ratio**	3:1	1:3	1:6	1:4	2:1	1:3

* After immortalization, ovarian cancer cells corresponding to type 1 and type 4 of primary cells were detected. Abbreviations: (+)—present, (−)—absent, E—Eosinophilic, B—Basophilic, N—nucleus, C—cytoplasm.

## Data Availability

All data generated or analyzed during this study are included in this article.

## Supplementary Materials

The following supporting information can be downloaded at: https://www.mdpi.com/article/10.3390/ijms25105384/s1.
