# Supplementary material for: Comparative Analysis of Primary Ovarian Cancer Cells and Established Cell Lines as a New Tool for Studies on Ovarian Cancer Cell Complexity
Source: Int J Mol Sci. 2024 May 15;25(10):5384. doi: 10.3390/ijms25105384 (PMC11121816; doi:10.3390/ijms25105384)
Supplement: Supplementary file 1 [file ijms-25-05384-s001.zip › Figure S1.pdf]

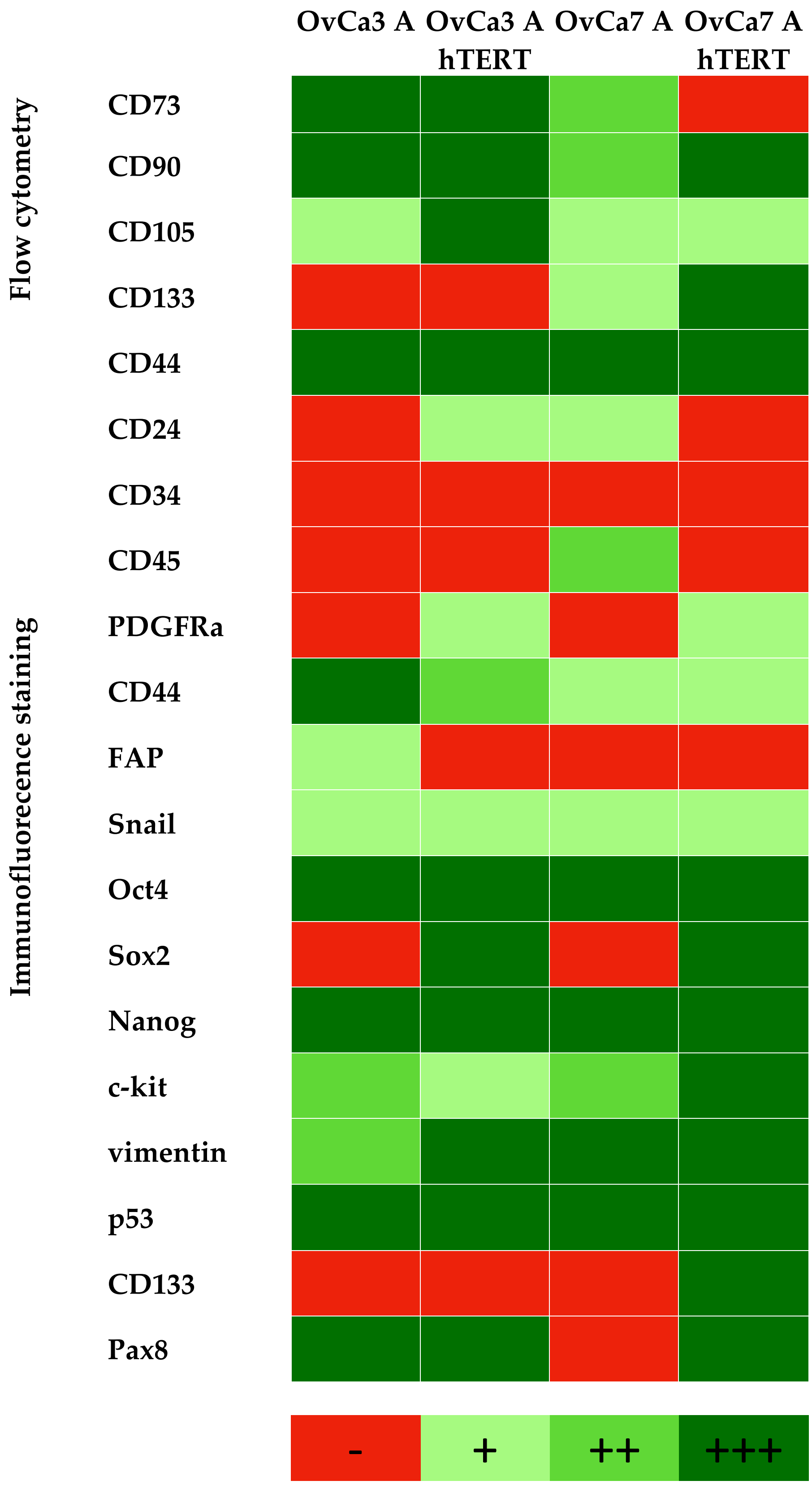

Figure S1. Heat map summarizing the phenotype characteristics of ovarian cancer cells before and after hTERT immortalization by using flow cytometry and immunofluorescence
