# Supplementary material for: Comparative Analysis of Primary Ovarian Cancer Cells and Established Cell Lines as a New Tool for Studies on Ovarian Cancer Cell Complexity
Source: Int J Mol Sci. 2024 May 15;25(10):5384. doi: 10.3390/ijms25105384 (PMC11121816; doi:10.3390/ijms25105384)
Supplement: Supplementary file 1 [file ijms-25-05384-s001.zip › Figure S2.pdf]

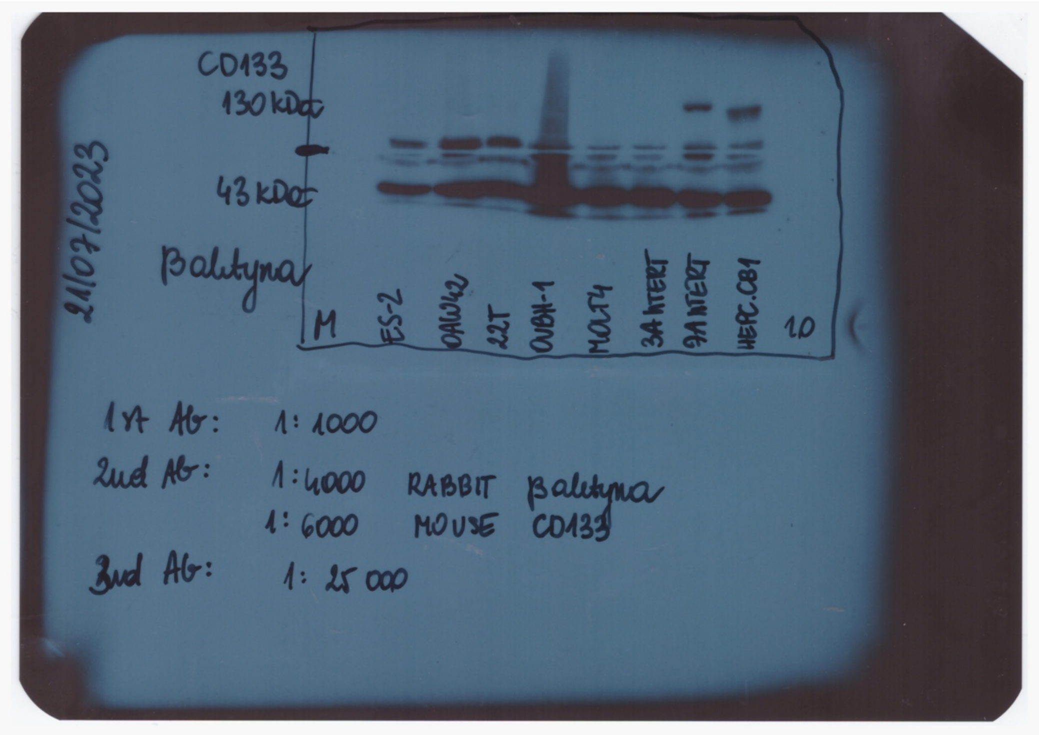


Figure S2. Western blot analysis of CD133 molecule expression in immortalized ovarian cancer cell lines. CD133 and β-Actin protein levels were revealed with different sets of specific antibodies in cell extracts obtained from lysing cells with RIPA buffer. Protein extracts from HEPC-CB.1 cells were used as a positive control CD133 – 130 kDa and β-Actin – 43 kDa expression. Each time 50 μg/line of total protein was loaded.
