# Supplementary material for: Comparative Analysis of Primary Ovarian Cancer Cells and Established Cell Lines as a New Tool for Studies on Ovarian Cancer Cell Complexity
Source: Int J Mol Sci. 2024 May 15;25(10):5384. doi: 10.3390/ijms25105384 (PMC11121816; doi:10.3390/ijms25105384)
Supplement: Supplementary file 1 [file ijms-25-05384-s001.zip › Figure S3.pdf]

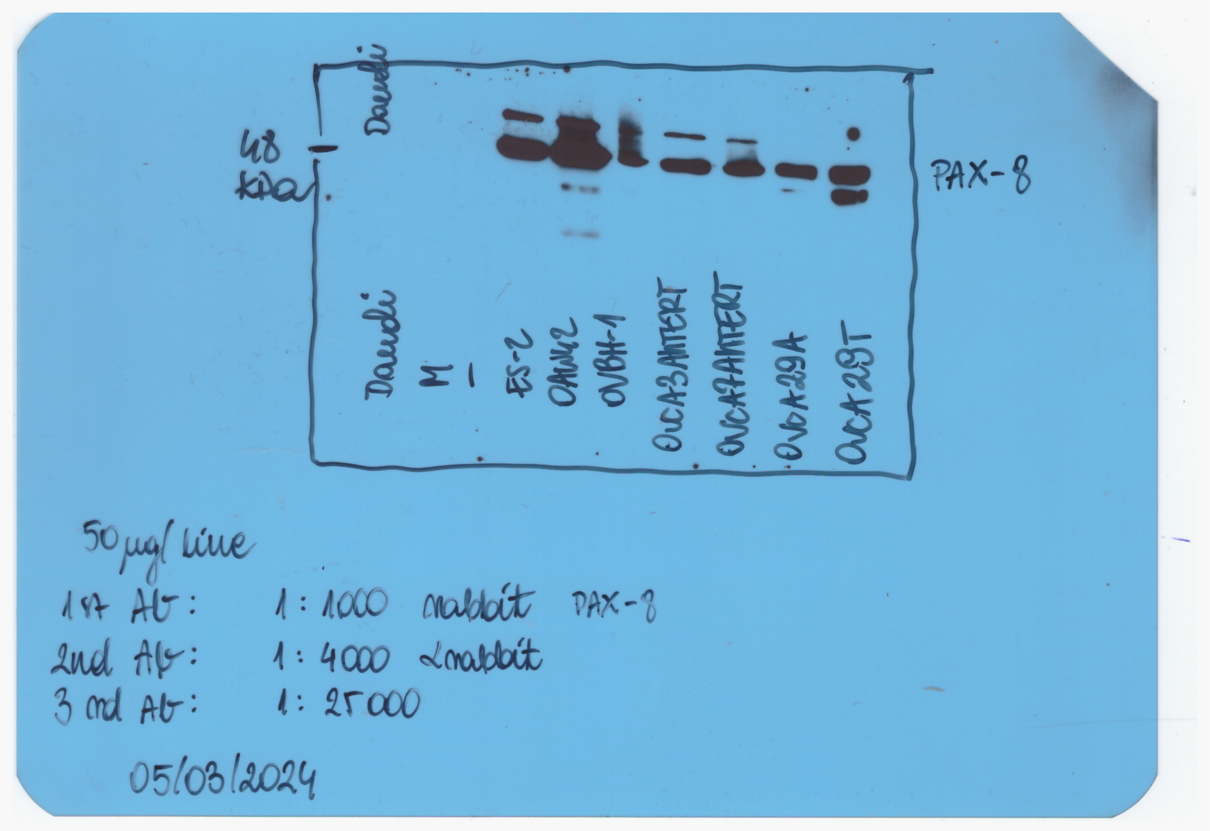

Figure S3. Western blot analysis of Pax8 molecule expression in immortalized cell lines. Pax8 and β-Actin protein levels were revealed with different sets of specific antibodies in cell extracts obtained from lysing cells with RIPA buffer. Protein extract from OAW-42 cells was used as a positive control, while protein extract from Daudi cells was used as a negative control. Pax8 –48 kDa and β-Actin – 43 kDa expression. Each time 50 µg/line of total protein was loaded.
