# Supplementary material for: Comparative Analysis of Primary Ovarian Cancer Cells and Established Cell Lines as a New Tool for Studies on Ovarian Cancer Cell Complexity
Source: Int J Mol Sci. 2024 May 15;25(10):5384. doi: 10.3390/ijms25105384 (PMC11121816; doi:10.3390/ijms25105384)
Supplement: Supplementary file 1 [file ijms-25-05384-s001.zip › Table S1 and S2.pdf]

Table S1. List of primary antibodies

Antigen	Host	Class of antibody	Clone	Company	Dilution	Incubation
Human PDGFRa	Mouse	Monoclonal	C-9	Santa Cruz	1:100	1h, Room Temperature (RT)
Human CD44	Rabbit	Polyclonal		Thermo Fisher Scientific	1:100	1h, RT
Human Fibroblast activation protein (FAP)	Mouse	Monoclonal	F11-24	Thermo Fisher Scientific	1:100	Overnight (O/N), $4^{0}C$
Human Snail1	Rabbit	Polyclonal		Bio Site	1:100	O/N, $4^{0}C$
Human Oct4	Rabbit	Polyclonal		Thermo Fisher Scientific	1:100	O/N, $4^{0}C$
Human Sox2	Mouse	Monoclonal	20 G5	Thermo Fisher Scientific	1:100	O/N, $4^{0}C$
Human Nanog	Rabbit	Polyclonal		Thermo Fisher Scientific	1:100	O/N, $4^{0}C$
Human vimentin	Mouse	Monoclonal	V9	Thermo Fisher Scientific	1:100	1h, RT
Human c-kit (CD177)	Mouse	Monoclonal	K45	Thermo Fisher Scientific	1:50	O/N, $4^{0}C$
Human p53	Mouse	Monoclonal	DO-7	Dako	1:50	1h, RT
Human CD133	Mouse	Monoclonal	2F8C5	Thermo Fisher Scientific	1:100	O/N, $4^{0}C$
Human Pax8	Rabbit	Monoclonal	D2S2I	Cell Sygnaling Technology	1:100	1h, RT
Human CA125	Mouse	Monoclonal	185 1	Leica Biosystem	1:100	1h, RT
Human Cytokeratin 8	Mouse	Monoclonal	TS1	Novocastra	1:100	1h, RT

Table S2. List of secondary antibodies

Antigen	Host	Class of antibody	Flouorophore	Company	Dilution	Incubation
F-actin		Phalloidin	Alexa Fluor 488™	Thermo Fisher Scientific	1:400	45min, RT
Mouse IgG	Goat	Polyclonal	Alexa Fluor 488™	Thermo Fisher Scientific	1:700	45min, RT
Mouse IgG	Goat	Polyclonal	Alexa Fluor 647™	Thermo Fisher Scientific	1:700	45min, RT
Rabbit IgG	Goat	Polyclonal	Alexa Fluor 647™	Thermo Fisher Scientific	1:700	45min, RT
